# The mechanopathology of the tumor microenvironment: detection techniques, molecular mechanisms and therapeutic opportunities

**DOI:** 10.3389/fcell.2025.1564626

**Published:** 2025-03-18

**Authors:** Stella Angeli, Constantina Neophytou, Maria Kalli, Triantafyllos Stylianopoulos, Fotios Mpekris

**Affiliations:** Cancer Biophysics Laboratory, Department of Mechanical and Manufacturing Engineering, University of Cyprus, Nicosia, Cyprus

**Keywords:** mechanical forces, tumor microenvironment, tissue stiffness, mechanopathology, cellular mechanotransduction, computational modelling

## Abstract

The mechanical properties of the tumor microenvironment (TME) undergo significant changes during tumor growth, primarily driven by alterations in extracellular (ECM) stiffness and tumor viscoelasticity. These mechanical changes not only promote tumor progression but also hinder therapeutic efficacy by impairing drug delivery and activating mechanotransduction pathways that regulate crucial cellular processes such as migration, proliferation, and resistance to therapy. In this review, we examine the mechanisms through which tumor cells sense and transmit mechanical signals to maintain homeostasis in the biomechanically altered TME. We explore current computational modelling strategies for mechanotransduction pathways, highlighting the need for developing models that incorporate additional components of the mechanosignaling machinery. Furthermore, we review available methods for measuring the mechanical properties of tumors in clinical settings and strategies aiming at restoring the TME and blocking deregulated mechanotransduction pathways. Finally, we propose that proper characterization and a deeper understanding of the mechanical landscape of the TME, both at the tissue and cellular levels, are essential for developing therapeutic strategies that account for the influence of mechanical forces on treatment efficacy.

## 1 Introduction

The mechanical characteristics of cells and tissues play a key role in shaping their structure, composition, and function. Changes in these properties are linked to various diseases, including cancer. Healthy cells and tissues undergo a number of alterations to become diseased and especially tumorigenic (Langley and Fidler, 2007; Lu et al., 2012). Research from various fields, including soft matter biophysics and mechanobiology, underscores the importance of studying cellular and tissue mechanics in tumors to understand cancer’s molecular structure and organization of their microenvironment (Kumar and Weaver, 2009; Wirtz et al., 2011). Importantly, the tumor microenvironment (TME) and molecular signalling have a great impact on the initiation, progression, drug resistance, and metastasis of tumors (Paszek et al., 2005; Tang et al., 2010; Liu et al., 2012a; Pickup et al., 2014; Tan et al., 2014; Chin et al., 2016; Hassell et al., 2017; Pfeifer et al., 2017; Sontheimer-Phelps et al., 2019; van Oosten et al., 2019; Nia et al., 2020).

It has been demonstrated that various mechanical properties of cancer cells experience changes during tumor progression, such as cellular tension (Tinevez et al., 2009; Logue et al., 2015; Logue et al., 2018; Adams et al., 2021), hydrostatic pressure (Logue et al., 2015; Logue et al., 2018; Adams et al., 2021), adhesion force (Sun et al., 2019; Massey et al., 2020), tissue elasticity and viscosity (Sun et al., 2019; Massey et al., 2020; Cartagena-Rivera et al., 2015; Krisenko et al., 2015; Efremov et al., 2018; Parvini et al., 2022). These changes play a significant role in the altered behaviour of cancer cells and cancer tissues. The mechanical deformation of cancer cells, driven by intracellular and intercellular forces within the TME, can activate mechanosensitive biochemical signalling pathways. These pathways then alter the cells’ molecular regulation and mechanical properties, which in turn can enhance their ability to metastasize (Kumar and Weaver, 2009). Mechanotransduction is the term that describes numerous processes wherein cells react to mechanical cues and physical forces to initiate intercellular signalling pathways that modulate proliferation, migration, invasion and survival. These processes play a vital role in cancer due to their contribution in tumor progression, metastasis and therapy resistance. Mechanical cues, such as the extracellular matrix (ECM) stiffness and mechanical forces, i.e., solid stress, interstitial flow shear stress, mechanical stretch and strain, are key factors of the TME. The complexity of the TME, comprising a dynamic interplay between cancer cells, stromal tissue, the ECM and the tumor vasculature significantly contributes to the challenges of effective cancer treatment. In this review, we describe how the development of mechanical forces during tumor progression leads to mechanotransduction and activation of various signalling pathways. Furthermore, we mention the existing strategies to target tumor mechanics and how emerging technologies and computational modeling serve as crucial tools for studying mechanical aspects of cancer.

## 2 Generation of mechanical forces during tumor progression

### 2.1 Elevation of solid stress and tissue stiffness impacts the tumor microenvironment

Highly proliferative cancer cells remodel the ECM to create a microenvironment that supports their survival, growth, and spread. Solid tumors and their associated TME consists of cancer cells and stromal components, including the ECM, basement membrane, blood vessels, immune cells, and fibroblasts. During tumor progression, these components undergo changes in both their structure and function producing solid stresses, i.e., mechanical stresses due to structural components of the tumor (Pickup et al., 2014; de Visser et al., 2006; Nyga et al., 2021). Cancer cells or activated fibroblasts are responsible for remodelling the ECM by stretching the collagen fibers, which along with the rapid growth of the tumor within the confined space of the host tumor, results in the generation of solid stress. Also, the activation of fibroblasts causes the secretion of transforming growth factor-β (TGFβ) (Casey et al., 2008; Costea et al., 2013; Papageorgis and Stylianopoulos, 2015; Pankova et al., 2016; Afik et al., 2016), which leads to an overabundance of ECM components including collagen, fibronectin, and hyaluronan, as well as increased collagen crosslinking. Overexpression of TGFβ is associated with increased Cancer Associated Fibroblasts (CAFs) elongation, cell spreading, lamellipodia formation and spheroid invasion (Stylianou et al., 2018a). The effects of TGFβ, make ECM stiffer, which in turn influences the behaviour of tumor cells (Kalli and Stylianopoulos, 2024). Increased tissue stiffness also triggers the activation of fibroblasts and the development of α-smooth muscle actin (α-SMA)-positive myofibroblasts (CAFs) (Stylianou et al., 2019). This creates a positive feedback loop in which enhanced CAFs activation drives ECM production, crosslinking, and myofibroblast contractility, leading to further stiffening of the tissue (Tomasek et al., 2002).

While myofibroblast-like CAFs are typically seen as promoters of tumor progression (Erez et al., 2010; Su et al., 2018), research has shown they can also have tumor-suppressive effects (Ozdemir et al., 2014; Rhim et al., 2014), depending on the type of fibroblast and its tissue of origin. For instance, a study showed that hyaluronan secreted by CAFs promoted tumor growth, while collagen-I secreted by CAFs had a tumor-suppressive effect (Bhattacharjee et al., 2021). Interestingly, the composition of immune cells in the TME either promote or inhibit ECM remodelling and, consequently, affect tissue stiffness. For instance, in mouse breast tumors, the elevated matrix stiffness was correlated with the accumulation of M2-like tumor-associated macrophages (TAMs) (Taufalele et al., 2023), while other immune cells (like cytotoxic T-cells) might contribute to ECM degradation. Consequently, a collagen-dense, rigid ECM limits T-cell infiltration and weakens their cytotoxic function. The increased stiffness creates a physical barrier for T-cells to penetrate the tumor, and it also diminishes their ability to effectively target and kill tumor cells (Reis-Sobreiro et al., 2021; Marofi et al., 2022; Vignali et al., 2023).

In accordance to these adaptations of the ECM, solid malignant tumors are mostly stiffer compared to healthy tissues or even benign tumors, as observed in breast (Sinkus et al., 2007; Evans et al., 2012; Boyd et al., 2014; Acerbi et al., 2015), pancreatic (Carrara et al., 2018), liver (Shahryari et al., 2019) and prostate cancer (Rouviere et al., 2017). For instance, human breast tumors were five times stiffer than the host healthy tissue, and this increased stiffness was strongly linked to higher malignancy (Evans et al., 2010), while in mice, mammary tumor tissue was 24 times stiffer than normal mammary tissue (Paszek et al., 2005). Additionally, the stiffness of human liver tissue was positively associated with the risk of developing hepatocellular carcinoma (Mueller and Sandrin, 2010). Apart from the overall tumor stiffening, another key mechanical characteristic of tumor tissue is the variation in stiffness within the tumor itself with significant spatial differences in tissue stiffness in both breast and liver tumors (Mak et al., 2013). For instance, in breast tumor biopsies, the stiffness at the tumor periphery was seven times greater compared to the tumor core with stiffness more similar to that of healthy breast tissue (Plodinec et al., 2012).

Tumor stiffening accompanied by the buildup of solid stress within tumors, causes the compression of intratumoral blood vessels, disrupting normal blood flow and contributing to therapy resistance (Stylianopoulos et al., 2012; Stylianopoulos et al., 2013; Jain et al., 2014; Voutouri et al., 2014; Mpekris et al., 2015; Nia et al., 2016; Papageorgis et al., 2017; Polydorou et al., 2017; Stylianopoulos et al., 2018; Panagi et al., 2020). Extensive collapse of blood vessels within tumors significantly reduces blood flow, a state referred to as hypoperfusion. This reduction in perfusion becomes a major obstacle for drug delivery, limiting the amount of therapeutic agents that can effectively reach the tumor. Furthermore, inadequate blood flow results in a hypoxic TME, which fuels tumor progression. Hypoxia not only allows cancer cells to evade immune detection but also promotes their ability to invade surrounding tissues and spread to distant sites (Helmlinger et al., 1997; Cheng et al., 2009; Samuel et al., 2011; Hidalgo and Von Hoff, 2012; Padera et al., 2004; Mpekris et al., 2017). In addition, hypoperfusion can limit immune cell infiltration into the tumor, while hypoxia creates an immunosuppressive TME, shifting TAMs from the immunosupportive M1 type to the immunosuppressive M2 type, and diminishing the cytotoxic capacity of effector immune cells (Panagi et al., 2020; Joyce and Fearon, 2015; Mariathasan et al., 2018; Wilson and Hay, 2011; Barsoum et al., 2014; Majidpoor and Mortezaee, 2021). Moreover, hypoxia fosters pro-tumor immune responses by increasing Programmed death-ligand 1 PD-L1 expression in myeloid derived suppressor cells (MDSCs), TAMs, dendritic cells (DCs), and cancer cells (Noman et al., 2014; Noman et al., 2015). It also attracts immunosuppressive T-regulator cells (Tregs) by triggering the expression of the chemokine CCL28 (Facciabene et al., 2011) and along with acidity (Calcinotto et al., 2012), affects differentiation and function of T-lymphocytes and myeloid cells (Palazon et al., 2012) and causes TAMs to promote angiogenesis (Laoui et al., 2014).

### 2.2 Development of fluid stresses during tumor progression

Fluid stresses–due to forces exerted by the fluid components of the tumor- are determined in large part by the combined effect of the structure of tumor vessels and the compression of blood and lymphatic vessels. Blood vessels formed during tumor angiogenesis become hyperpermeable, leading to excessive fluid leakage into the tumor’s interstitial space. Vessel hyperpermeability is characterized by an increase in the pore size of the vessel walls, primarily driven by elevated levels of pro-angiogenic factors, such as vascular endothelial growth factor (VEGF), which promote tumor-induced angiogenesis (Jain, 2014). The newly formed vessels during angiogenesis typically exhibit structural abnormalities, including disorganized vascular patterns, poor intercellular connections between endothelial cells, insufficient coverage by pericytes, and either a disrupted or absent basement membrane (Jain, 1987; Jing et al., 2019). The collapse or dysfunction of lymphatic vessels both within and around the tumor along with vessel hyperpermeability, result in fluid accumulation and increased interstitial fluid pressure (IFP) within the tumor (Padera et al., 2004). While in the core of the tumor IFP is elevated, in the periphery of the tumor IFP drops sharply to normal values. The pressure difference between the tumor core and periphery causes interstitial fluid flow towards the margin of the fluid that oozes growth factors, nutrients, small molecule chemotherapies from the tumor to the surrounding normal tissue that contributes to tumor progression (Polacheck et al., 2011). Furthermore, the high IFP eliminates transvascular pressure gradients and thus, can prevent effective delivery and distribution of large therapeutic agents, reducing the effectiveness of treatments like nanomedicine and immunotherapy (Jain, 2013).

### 2.3 Viscoelastic properties of tumors and their impact

In addition to tissue stiffening and IFP elevation, tumor tissues also exhibit distinct viscoelastic properties compared to normal tissues. Viscoelasticity in the TME refers to the combined mechanical properties of viscosity (resistance to flow) and elasticity (ability to return to shape after deformation) that characterize the behaviour of tissues within and around a tumor. Viscoelastic materials are deformed gradually over time when a constant external stress or load is applied and then relax as stress diminishes over time in response to a constant deformation (i.e., stress relaxation) (Chaudhuri et al., 2020).

The increased stiffness as described above contributes to TME’s altered viscoelastic properties, making it more resistant to deformation under stress (Streitberger et al., 2020). For instance, the fluidity of benign meningioma tissue was 3.6 times greater than that of aggressive glioblastoma tissue, whereas the more solid-like nature of glioblastomas contributed to their ability to aggressively infiltrate surrounding tissues (Streitberger et al., 2020). While viscoelasticity and stiffness are closely connected, a study demonstrated that changes in matrix viscoelasticity promoted liver tumor niche independently of stiffness (Fan et al., 2024), due to changes in the cellular shape, cytoskeletal reorganization and the formation of invadopodia-like structures (Chaudhuri et al., 2020; Wisdom et al., 2018; Adebowale et al., 2021). The viscoelastic properties of the tumor contribute to stress relaxation which is also linked to poroelastic deformation. In solid tumors, elevated ECM fiber density, anisotropy and cell volume apply forces on the matrix that control the viscous fluid flow, resulting in local deformations in pore volume. Also, a component of viscoelasticity is mechanical plasticity, where the matrix experiences permanent deformations when the applied stress surpasses its yield stress leading to tumor cell migration (Wisdom et al., 2018). Moreover, hyaluronan, which has a strong negative charge that enables it to attract and trap water molecules, leads to significant hydration and swelling. This characteristic becomes particularly pronounced in tumors with high levels of hyaluronan, where the accumulation of water creates a swelling pressure within the tissue (Voutouri and Stylianopoulos, 2014; Voutouri et al., 2016). This hyaluronan-derived swelling pressure not only increases the IFP but also contributes to the viscoelastic properties of the tumor tissue.

The physical properties of the TME as described above profoundly impact not only nutrient and drug delivery but also cellular behaviour and signalling. The heterogeneous nature of TME generates mechanical signals which arise from alterations in the tumor tissue and interactions with surrounding cells and structures. These structures are called mechanosensors and they are the gatekeepers of tumor dynamics.

## 3 Mechanosensors and cellular mechanotransduction in cancer

**FIGURE 1:**
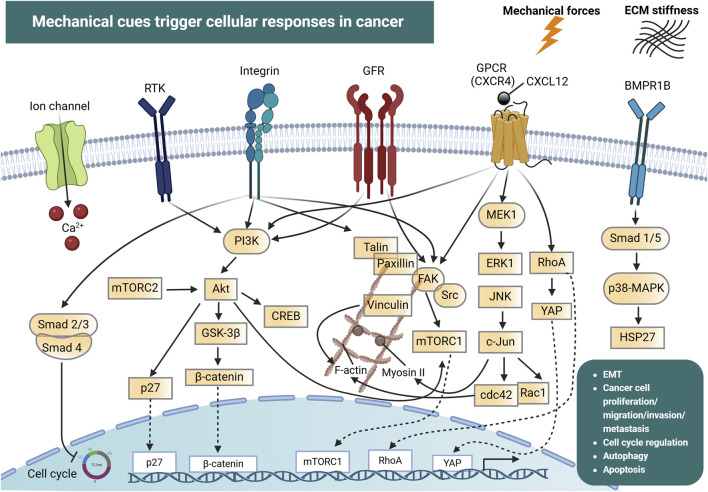
Mechanical cues and cellular mechanotransduction pathways in cancer. Mechanical forces together with extracellular matrix (ECM) stiffness are detected by ion channels (e.g., Piezo), integrins, G-protein coupled receptors (GPCRs) and the bone morphogenic protein receptor 1B (BMPR1B). In addition, receptor tyrosine kinases (RTKs) and growth factor receptors (GFRs) can be activated by mechanical forces in the tumor microenvironment. This drives the initiation of signal transduction pathways including PI3K, FAK, p38-MAPK and JNK, which interact in a highly intricate manner and regulate processes such as cell cycle progression, cytoskeletal reorganization (mediated by F-actin, Myosin II, RhoA, cdc42), epithelial-mesenchymal transition (EMT), cancer cell proliferation, migration, invasion and metastasis. These force-induced reactions can also drive the nuclear translocation of transcription factors which directly modulate the expression of genes involved in cancer development and progression. Created with BioRender.com.

At the cellular level, mechanosensors can detect mechanical forces from the extracellular space and convert them into biochemical signals through mechanotransduction pathways (Figure 1). Cell surface mechanosensors include integrins, cadherins (Hoffman and Yap, 2015; Driscoll et al., 2021; Sun et al., 2022), G-protein coupled receptors (GPCRs) (Langenhan, 2020) and ion channels (Karska et al., 2023).

Integrins and cadherins are transmembrane receptors that link the ECM to the cytoskeleton and are responsible for mediating cell adhesion and transmitting chemical signals to the cell interior via catch bonds (Cooper and Giancotti, 2019; Mierke, 2024). The transmission and distribution of forces through these adhesion receptors, are critically influenced by factors like the rate of force application, and the spatial distribution of the applied force (Elosegui-Artola et al., 2018). As mentioned above, during cancer progression, cells are exposed in high mechanical forces and can also exert forces on their surrounding environment (Mierke, 2024). Intracellular forces can be generated in the presence of a stiff extracellular environment, due to cell spreading and cytoskeletal re-organization, leading to the development of high traction forces (Sun et al., 2021). Forces that exceed a certain level can induce conformational and organizational modifications in groups of proteins that act as mechanosensors. Some of these proteins are talin, vinculin, focal adhesion kinases (FAK), integrins and stretch-sensitive ion channels (Mierke, 2024). Such conformational alterations allow these proteins to act as scaffolds for the recruitment of new structural proteins, which enhances force transmission, leading to the conversion of mechanical signals into biochemical signals, which in turn determines cellular responses. For example, the actomyosin complex generates sufficient traction to balance intracellular and extracellular forces via adhesive proteins (Sun et al., 2021). This traction force propagates along actin filaments toward the nucleus, influencing gene expression and ultimately dictating cell fate.

Research groups over the years have been investigating how cancer cells respond to mechanical forces and their microenvironment under controlled conditions, through various *in vitro* experimental designs, offering insights into cancer progression and metastasis. Findings from such experiments provided clarity on the mechanotransduction mechanisms employed by cancer cells. These experimental protocols primarily consist of transmembrane pressure devices used to apply a defined compressive force on a 2D cell monolayer, and 3D cell cultures, where cells are grown individually or as spheroids embedded in hydrogels or enclosed within microcapsules (Kalli and Stylianopoulos, 2024). These experiments replicate solid and fluid stresses to simulate the mechanical environment experienced at the cellular level. An overview of various mechanosensor molecules and mechanotransduction signalling pathways is summarized in Table 1, highlighting the intricate mechanisms by which cells detect and react to mechanical forces in their microenvironment, as validated in *in vitro* experiments. Such pathways play critical roles in cancer pathology by regulating processes, such as tumor progression, proliferation, apoptosis, migration and invasion.

**TABLE 1:** Main molecular signalling pathways and mechanosensors involved in cellular responses to mechanical stresses in cancer and approaches to alleviate them.

Signalling pathway/Mechanosensors	Effect in TME	Effect in cancer progression	Cancer type	Targeted proteins	Inhibitors/Mechanotherapeutic agents	References
Piezo ion channel upregulation/Calcium signalling	Immunomodulation; Enhance ECM remodelling	Regulate tumor cell behaviour; Activation of apoptosis; Cell migration, proliferation and invasion; Matrix degradation; Metastasis	Gastric; Breast; Prostate; Glioma; Osteosarcoma; Synovial sarcoma; Bladder	Calcium ions	Gallolinium; Ruthenium red; GSMTx4 peptide	Wu et al. (2017), Prevarskaya et al. (2014), Zhang et al. (2018b), Li et al. (2015), Etem et al. (2018), Zhang et al. (2024), Luo et al. (2022), Pardo-Pastor et al. (2018), Han et al. (2019), Yang et al. (2016a), Chen et al. (2018), Jiang et al. (2017), Suzuki et al. (2018), Jiang et al. (2022)
FAK	Promote vascular and ECM remodelling; Prevent immune evasion	Promote cell survival and proliferation; Enhance cell invasion and metastasis; Tune mechanotransduction	Breast; Ovarian; Pancreatic; Lung; Melanoma; Prostate; Colorectal; Glioblastoma; Esophageal cancer	Talin; Vinculin; Paxillin; p130Cas; Caveolin 1	BI-853520 (IN10018); GSK2256098; NVP-TAC544; PF-431396; PF-573228; TAE226; VS-4718; VS-6062 (PF-00562271); Avutometinib - Defactinib (VS-6063); 1H-Pyrrolo(2,3-b) pyridine; APG-2449	Chuang et al. (2022), NCT03917043UNLoMCg (2023)
Notch	Regulate EMT; Shape tumor vasculature and immunity	Promoting cell proliferation, invasion and chemoresistance	Breast; Hepatocellular carcinoma; Colorectal; Prostate	Snail; Slug; Twist; Cyclin D1	Gamma-secretase inhibitor (MK-0752, siRNA (MEDI0639) Synthetic Notch receptor; SynNotch	Yuan et al. (2014), Meurette and Mehlen (2018), Sloas et al. (2023)
PI3K/AKT	Increase vascular permeability and angiogenesis; Promote actin remodelling; Changes in the cytoskeleton that enhance cell migration; Macrophages polarization toward a M2-like phenotype	Promote cell growth and resistance to apoptosis; Favouring glycolysis and lipid synthesis that support energy demands of growing tumors; Increase migratory potential	Colorectal; Melanoma; Breast; Renal cell carcinoma; Esophageal squamous cell carcinoma; Hepatocellular carcinoma; Gastric; Prostate; Pancreatic	PI3K; mTORC1; mTORC2; AKT	Alpelisib; Copanlisib	Kalli et al. (2019a), Xu et al. (2020b), Mafi et al. (2021), Liu et al. (2018), Xu et al. (2021), Yan et al. (2020)
Wnt-b-catenin and RET-b-catenin	Promote; ECM remodelling; Angiogenesis and immune evasion	Excessive tumor cell proliferation; EMT, migration and invasion; Infude chemoresistance	Colorectal; Hepatocellular carcinoma; Breast	β-Catenin; c-Myc; Cyclin D1; Axin2; Matrix metalloproteinases; RET; Frizzled receptors; Dickkopf Wnt signalling pathway inhibitor 1	DKN-01; Aspirin; Selumetinib - cyclosporine	NCT04363801UNLoMCg (2024), Joharatnam-Hogan et al. (2019), Krishnamurthy et al. (2018)
Yes-associated proteins (YAP)-TAZ	Regulate the behavior of CAFs contributing in ECM remodeling; Angiogenesis and immune suppression	Activation of pro-cancerous genes	Pancreatic; Breast	YAP; TAZ; TEAD1; TEAD2; TEAD3; TEAD4; LATS1; LATS2	Simvastatin - cerivastatin; Simvastin - anti-HER2); Verteporfin/Visudyne;	Jiang et al. (2022), Ortega et al. (2021), Hao et al. (2019), Ricco and Kron (2023), NCT03324425UNLoMCg (2020)
TGFβ	Increase activation, differentiation and proliferation of CAFs and ctivate CAF-mediated matrix stiffening; Promote expression of MMPs to degrade ECM; Induce immunosuppression; Stimulates angiogenesis.	Promote EMT; Facilite tumor invasion;	Breast; Non-small cell lung cancer; Colorectal; Prostate; Head and Neck; Pancreatic; Glioblastoma; Osteosarcoma	TGFβ; SMADs; TGFβR1; TGFβR2	Pirfenidone; Losartan; Tranilast	Papageorgis et al. (2017), Polydorou et al. (2017), Panagi et al. (2020), Mpekris et al. (2021), Panagi et al. (2022), Mpekris et al. (2023a), Chauhan et al. (2013), Diop-Frimpong et al. (2011), Liu et al. (2012b)
Src signalling pathway	Actin cytoskeleton reorganization; Enhance cell adhesion	Increase invasive potential and Metastasis	Lung; Breast; Pancreatic; Colon; Prostate	B1-integrin; Vinculin; FAK; F-actin; Paxillin	Dasatinib; AZD0530; SKI-606	Thamilselvan and Basson (2004), Kim et al. (2009)
CXCR4/CXCL12 signalling	Chemokine-mediated autologous chemotaxis	Increase invasion	Hepatocellular carcinoma; Ovarian; Renal cell carcinoma	CXCR4; CXCL12; Integrins (α4β1, α5β1)	AMD3100	Shah et al. (2015), Ruiz et al. (2018)
MEK/ERK1 pathway	Promote ECM remodelling; Promote growth factor secretion	Increase invasion; Induce cell migration and tumor aggressiveness	Hepatocellular carcinoma; Glioblastoma	RAF, MEK1-2; ERK1; ERK2	Avutometinib - defactinib	Yoshimura et al. (2024)
mTOR	Invadopodia formation; MT1-MMP expression; ECM degradation	Increase metastasis	Breast; Pancreatic; Servical; Non-small cell lung cancer; Colon	FKBP12; FRB	Rapamycin; Allosteric inhibitors R1-R5	Yang et al. (2016b), Mao et al. (2022)
P38-MAPK	Activate CAFs to produce ECM components; Stimulate new blood vessel formation; Activate pro-inflammatory cytokines	Increase cell migration; Increase EMT markers; Formation of filopodia and stress fibres; Regulate proliferation upon compression	Breast; Brain; Pancreatic; Prostate	P38; MAPK; MK2	SB-242235	Yang et al. (2024)
JNK signalling	Cytoskeletal reorganization	Increases cell migration; Increase EMT markers; Formation of filopodia and stress fibres; Regulate proliferation upon compression	Pancreatic; Lung; Breast; Skin; B-lymphoma; Osteosarcoma	Rac1; cdc43; myosin II; JNK1; JNK2; JNK3; JUN	PYC98 and PYC71N inhibitors	Wu et al. (2020)
Rho-ROCK RhoA)	Promote actin stress fiber formation; Promote EMT; Promote myofibroblast differentiation; Activate CAFs to produce collagen and fibronectin; Increase vascular permeability; Induce immune response modulation	Promote contractility and cell migration leading to tumor invasion and metastasis; Mediate the invasive and proliferative properties of tumor cells.	Osteosarcoma Liver; Hepatocellular carcinoma, Colorectal; Squamous cell carcinoma; Head and neck squamous cell carcinoma; Glioblastoma; Thyroid carcinoma; Lung adenocarcinoma; Prostate; Testicular germ cell tumors; Melanoma; Breast; Gastric; Pancreatic; Endometrial carcinoma; Ovarian cancer; Bladder Urothelial carcinoma	ROCK1; ROCK2; LIM kinase; Cofilin; FAK; NF-kB; YES kinase; TAZ/YAP; Vimentin	Rock inhibitors (Y-27632 and fasudil); AT13148; FAK inhibitors (e.g.,.Defactinib, PF-562271); Integrin inhibitors (Cilengitide); LIM kinase inhibitors (LimKi-1)	Chuang et al. (2022), Barbazan et al. (2023), Boyle et al. (2020), Crosas-Molist et al. (2022), McLeod et al. (2020), Chin et al. (2015), Lee et al. (2019)
Angiotensin Receptor 1 (AGTR1)	Promote sodium retention in the TME affecting tumor IFP; Promote vascular remodeling and angiogenesis; Induce production of collagens and hyaluronan by CAFs	Promote angiogenesis; Promote tumor growth and invasion; Promote immune evasion.	Pancreatic; Glioblastoma; Hepatocellular carcinoma	VEGFR; Angiopoietin 1&2; TGF-β1; Connective tissue growth factor; Endothelin-1	Losartan	Chauhan et al. (2013), Diop-Frimpong et al. (2011), Liu et al. (2012b)

### 3.1 Mechanical forces effects on cellular responses

The restriction of physical space around the tumor due to the presence of ECM components or a dense microenvironment was determined as the key factor for the generation of solid stress in the TME (Cheng et al., 2009). Solid stress found to have a significant impact on cancer cell proliferation, as demonstrated in breast and colon tumor spheroids growing within an agarose matrix, free from other ECM components (Helmlinger et al., 1997; Cheng et al., 2009). Evidence showed that solid stress can also regulate colon cancer cell division when cells grew as spheroids in a Dextran matrix of increasing concentrations (Montel et al., 2012). Furthermore, by utilizing polydimethylsiloxane (PDMS), which is a synthetic polymer that mimics the ECM, it was observed that mitotic division of colorectal tumor cells growing in spheroids was interrupted (Desmaison et al., 2013).

The impact of solid stress was also shown in cell migration and invasion. The mechanically driven migration of breast cancer cells showed the presence of actomyosin contractility and the formation of specialized cells at the forefront, which act as leader cells enabling directional migration (Tse et al., 2012). The generation of leader cells has been observed to be a result of Notch signalling activation, triggered by mechanical stress (Riahi et al., 2015). Also, breast and glioma cancer cells growing in an agarose 3D matrix showed metastatic characteristics under compressive forces, as revealed by their gene expression profiles (Demou, 2010). Epithelial-mesenchymal transition (EMT) was also facilitated in renal carcinoma cells, triggered by compressive forces, induced by increased levels of interleukin-6 (IL-6) via the AKT/GSK-3b/b-catenin pathway activation (Chen et al., 2017). Under the effect of solid stress, the activation of the PI3K/AKT signalling pathway in pancreatic cells also supported their increased migratory capabilities (Kalli et al., 2019a). Compressive forces that increased cell mobility were shown to activate the MEK1/ERK1 signalling pathway in glioblastoma cancer cells and facilitate interactions between microRNA (i.e., mir-548) and mRNA (Kalli et al., 2019b; Calhoun et al., 2020). In addition, overexpression of vascular endothelial growth factor A (VEGFA) in breast cancer cells due to mechanical compression, was induced via DNA methyltransferase 3 alpha (DNMT3A)- dependent miR-9 reduction (Kim B. G. et al., 2017). Fuentes-Chandia et al. (2021) showed the activation of malignant responses in breast cancer cells within spheroids due to compressive forces. These cells were characterized by stemness, showed migrative and proliferative features, enhanced contractility and proteolytic ability, via the secretion of matrix metalloproteinases (MMPs). Also, mechanical compression of ovarian cancer cells grown within 3D hydrogels, was found to foster proliferation, invasion and chemoresistance through the expression of cell division cycle 42 (cdc42), a key molecule that regulates the cytoskeleton (Novak et al., 2020). In addition, compressive stress was shown to trigger cell cycle arrest mediated by cyclin dependent kinase inhibitor, p27 (Delarue et al., 2014).

Tumor cells in viscoelastic environments were more motile and exhibit higher levels of vimentin expression and lower levels of cytokeratin, which boost their migratory ability through the EMT process (Elosegui-Artola et al., 2023). Additionally, in contrast to purely elastic substrates, viscoelasticity influenced stress fiber formation and promoted the nuclear translocation of Yes-associated proteins (YAP) serving as an oncoprotein, a process that is positively associated with tumor metastasis and chemoresistance (Wei et al., 2018; Zhang X. et al., 2018).

Fluid shear stress was found to disrupt cancer cell cycle progression by altering the expression levels of various cyclins, such as cyclin B1 and p21. This effect was mediated through integrin-dependent activation of Smad signalling pathways, leading to cell cycle arrest (Chang et al., 2008). This mechanotransduction pathway facilitates processes such as EMT, cell invasion, and tumor progression. For instance, the interplay between integrins and the TGF-β pathway, mediated by Smad signalling, is observed in various cancers (Papageorgis et al., 2010; Papageorgis et al., 2011). This interaction can enhance cellular responses to mechanical stimuli, contributing to changes in cell adhesion, migration, and proliferation, which are crucial for metastasis. The dysregulation of this axis, often driven by tumor-associated changes in the ECM and mechanical stress, exemplifies the critical role of mechanotransduction in tumor biology (Li et al., 2023; Sun et al., 2023; Rahman et al., 2024).

### 3.2 Tumor motility and invasion: integrin and focal adhesion signalling

Various signalling pathways that control cytoskeletal dynamics, adhesion, and motility are found to be activated by mechanical stresses during tumor progression. Such a pathway is the Src, which is a non-receptor tyrosine kinase that is activated by transmembrane protein receptors including integrins and GPCRs, leading to increased invasive potential and metastasis of cancer cells (Sun Z. et al., 2016). Activation of Src was shown to be regulated by mechanical signals in colon cancer cells, and specifically by non-laminar shear stress, leading to actin cytoskeleton reorganization and enhanced adhesion of cells to a collagen I substrate (Thamilselvan and Basson, 2004). Also, when interstitial fluid flow was applied to breast cancer cells cultured within a 3D collagen I scaffold, it activated β1-integrin, leading to the recruitment and activation of key signalling molecules such as vinculin, FAK, F-actin, and paxillin. This activation promoted the formation of cellular protrusions that guided the directional migration of the cells. Furthermore, the flow-induced migratory response was also mediated by the transmembrane C-C chemokine receptor 7 (CCR7), which, like β1-integrin, activated the FAK signalling pathway (Polacheck et al., 2011). FAK is a key integrator of mechanical forces, particularly in the context of cell adhesion to the ECM. It plays a key role in regulating the dynamics of integrin-based cell adhesions, which are essential for cellular motility. When integrins engage with ECM components, mechanical forces like shear stress or stretch activate FAK, which in turn triggers downstream signalling cascades that promote cytoskeletal reorganization, cell migration, and invasion. Experimental studies revealed that FAK promoted 3D matrix invasion by enhancing cellular stiffness and facilitating the transmission of actomyosin-dependent contractile forces within dense 3D extracellular matrices, in breast cancer cells (Mierke et al., 2017). Moreover, the FAK pathway was intricately linked with Src and paxillin, signalling molecules that further modulated cancer cell motility and survival (Chuang et al., 2022).

Enhanced motility induced by interstitial flow has also been demonstrated in glioma and hepatocellular carcinoma cells using 3D invasion assays. In hepatocellular carcinoma, interstitial flow promoted cell invasion towards the liver through the activation of CXCR4/CXCL12 signalling and the MEK/ERK pathway (Shah et al., 2015). Similarly, glioblastoma cells exhibited compression-induced invasion driven by MEK1/ERK1 pathway activation, highlighting the shared mechanotransduction pathways facilitating tumor progression in response to mechanical forces (Kalli et al., 2019b). CXCL12, the ligand of CXCR4 which is a chemokine secreted by stromal cells, created a gradient that directs CXCR4-expressing cancer cells toward specific sites, such as metastatic niches (Domanska et al., 2013). The upregulation of CXCR4 stimulated downstream pathways, like MEK/ERK and PI3K/AKT (Shi et al., 2020). These pathways influence cytoskeletal organization, cell adhesion processes, and cell survival (Fincham et al., 2000; Deng et al., 2022), further connecting CXCR4/CXCL12 signalling to mechanical cue integration within the TME. Moreover, it was proven that the migration of hepatocellular and liver carcinoma is increased, because of interstitial fluid shear stresses, created in a 2D parallel plate flow chamber, by the activation of integrin/FAK/RhoGTPase signalling pathway (Sun et al., 2018; Yu et al., 2018).

### 3.3 Piezo ion channels and calcium signalling

Regulators of cellular processes are also ion channels such as Piezo, which play a crucial role as mechanosensors in the translation of extracellular mechanical cues into intracellular calcium signals. Piezo proteins are the structural components of ion channels that respond to mechanical forces by opening and permitting the influx of positively charged ions, such as calcium into the cell (Coste et al., 2010; Wu et al., 2017). Physical forces affecting the plasma membrane, trigger these channels to facilitate calcium influx therefore activating downstream signalling pathways critical for tumor progression (De Felice and Alaimo, 2020). They play a key role in enhancing cancer cell adaptability within the mechanically dynamic TME, by modulating processes like cytoskeletal dynamics and invasion. The disruption of calcium homeostasis contributes to the enhancement of various cancer hallmarks, including apoptosis, cell migration, proliferation, invasion, and metastasis (Prevarskaya et al., 2014). Evidence of this has been demonstrated in many malignancies through both *in vitro* experiments and studies using real patient samples. *In vitro* studies have shown upregulation of Piezo1 and/or Piezo2 channels in gastric (Deng et al., 2022), breast (Sun et al., 2018; Yu et al., 2018; Coste et al., 2010), prostate (Wu et al., 2017), glioma (De Felice and Alaimo, 2020; Prevarskaya et al., 2014), osteosarcoma (Zhang J. et al., 2018), synovial sarcoma (Li et al., 2015), and bladder cancers (Etem et al., 2018). Additionally, analyses of human patient samples, such as those from Merkel cell carcinoma (Garcia-Mesa et al., 2022), bladder cancer (Etem et al., 2018), gastric cancer (Zhang et al., 2024), colon (Shang et al., 2023), and breast cancer (Martin-Sanz et al., 2024), further confirm their increased expression in clinical settings.

### 3.4 Tumor adaptability in mechanical stress via mTOR and PI3K pathways

Another component in the complex mechanotransduction cascade is the mechanistic target of rapamysin (mTOR), a key serine/threonine kinase, which integrates multiple signalling pathways to regulate cancer cell mechanics and transduce extracellular mechanical signals (Nazemi and Rainero, 2020; Di-Luoffo et al., 2021). The regulation of mTOR is influenced by upstream factors, such as membrane receptors, integrins, and components of focal adhesion complexes (Mousavizadeh et al., 2020). Kinases and phosphatases associated with mTOR, along with GTP-binding proteins and transcriptional regulators, play roles in pathways influenced by varying mechanical stimuli. Under nutrient-rich conditions, mTOR promoted anabolic activities such as the synthesis of proteins, lipids, and nucleotides, while concurrently suppressing the autophagic processes of the cell (Takahara et al., 2020). In addition, one of the two complexes comprising the mTOR, called the mTORC2, facilitated cell growth and survival by activating AKT, a key component downstream of growth factor signalling pathways (Fu and Hall, 2020). Pressure-stimulated mechanotransduction, such as shockwave stimulation was utilized to analyse the mTOR-FAK signalling (Lee et al., 2017). GSK-3β, AKT, and mTORC1 kinases were activated in response to the stimulation, however mTORC1 blocked FAK phosphorylation, suggesting that mTORC1 functioned as the primary regulator of shockwave-induced FAK phosphorylation. Evidence shows that mTOR activation increases actin stress fibers and mislocalizes mTORC1 to vesicle-like structures on microfilaments. This suggests mTORC1 and microfilaments regulate FAK phosphorylation and drive mesenchymal stem cell proliferation via mTORC1-FAK signalling (Mierke, 2024; Lee et al., 2017).

Among the pathways shown to be upregulated under the influence of vascular and interstitial shear forces is the PI3K pathway in breast cancer cells (Tchafa et al., 2015). This pathway drives critical processes such as cell growth, survival, migration, and metabolism (Hoxhaj and Manning, 2020). Mechanical cues can activate upstream mechanosensors like integrins and receptor tyrosine kinases, which in turn stimulate this pathway. In the context of mechanotransduction, PI3K activation enhances cytoskeletal remodelling and focal adhesion turnover, facilitating cancer cell motility and invasion (Deng et al., 2022). Additionally, this pathway intersects with other key mechanotransductive cascades, including the Akt and mTOR pathways, to support cellular adaptation to the mechanical properties of the TME (Glaviano et al., 2023). The PI3K/AKT/mTOR (PAM) pathway is a highly preserved signalling network that supports cellular survival, growth, and cell cycle progression. The PAM cascade is the most upregulated signalling pathway in human cancers and it is frequently associated with resistance to anticancer treatments. Additional evidence implicating the PI3K/AKT/CREB signalling pathway was highlighted by Kalli et al. (2019a), by utilizing a monolayer of pancreatic cancer cells in a 2D transmembrane pressure device and demonstrating their increased migration capabilities.

Upregulation in the P13K pathway was suggested to play a role in the interactions between cancer cells and CAFs (Feng et al., 2022). The effect of shear stress in a 2D parallel flow chamber, with a monolayer of liver cancer cells, caused the secretion of exosomes by liver cancer cells, followed by the activation of stellate cells, via PI3K signalling pathway. CAFs, are key players in the TME and it is suggested that PI3K signalling is often upregulated in CAFs and promotes their activation from quiescent fibroblasts. CAFs secrete growth factors, cytokines, and ECM components that support cancer cell survival and invasion. For example, PI3K activation in cancer cells stimulated the release of TGF-β and other signalling molecules, which in turn recruited and activated CAFs. This was observed by Shieh et al. (2011), who created a co-culture system consisting of breast cancer cells and fibroblasts in a collagen matrix. When interstitial flow was applied, they observed that fibroblast migrated along the collagen fibers. This effect was mediated by enhanced activation of TGF-β1 and collagen degradation, processes that collectively facilitated increased tumor cell invasion (Shieh et al., 2011).

### 3.5 Stress-induced cancer growth via P38 MAPK and JNK signalling

Another key pathway mediating mechanotransduction mechanisms is the p38 MAPK and JNK signalling. These kinases were found to drive proliferation, migration and invasion of cancer cells (Lin et al., 2005; Wagner and Nebreda, 2009; Taniuchi et al., 2015). Research has demonstrated that the activation of this pathway via mechanical compression or stress led to the initiation of cellular responses like autophagy, invasion, cytoskeletal reorganization and ECM remodelling (Blawat et al., 2020; Ong et al., 2020). Das et al. (2019) showed that under compression, HeLa cells cultured within alginate capsules, initiated autophagy and their invasive potential was increased, primarily through the activation of p38 MAPK signalling. Similarly, Lien et al. (2013) revealed the activation of this pathway and activation of autophagy in four cancer cell lines (i.e., hepatocarcinoma, osteosarcoma, oral squamous carcinoma and carcinomic alveolar basal epithelial cells), where interstitial shear stress was applied. More specifically, interstitial shear stress-induced cellular apoptosis and autophagy occurred via the activation of bone morphogenetic protein receptor type (BMPR) 1B/Smad1/5/p38 MAPK cascade. Also, the p38 MAPK/HSP27 and JNK/c-Jun pathways were shown to be essential for the proliferation and motility of pancreatic cancer cells under mechanical stress, as well as for the activation of Rac1, cdc42 and myosin II, which are responsible for cytoskeletal reorganization (Kalli et al., 2022).

Yan et al., supported that fluid shear stress stimulated liver cancer stem cell proliferation, enhanced the capacity of spheroid formation and facilitated migration through the RhoA-YAP1 autophagy pathway (Yan et al., 2022). It was found that phosphorylated Tyr42 (p-Tyr42) of RhoA, enabled RhoA to bind directly to the promoters of specific nuclear genes, thus regulating their expression and driving oncogenic processes (Kim J. G. et al., 2021; Zhan et al., 2022). Also, research showed that RhoA activation drove cancer cell invasion, metastasis and EMT induction in gastric cancer (Xu et al., 2020a; Zhang et al., 2020; Zhan et al., 2021). Furthermore, YAP is activated by RhoA, as shown in osteosarcoma and led to resistance to photodynamic therapy, due to increased tumor cell survival mechanisms (Zhan et al., 2021). GTPases like RhoA and Rac1 were shown to be activated in hepatocellular carcinoma cell lines, due to electromagnetic forces causing cytoskeletal reorganization, which is known to facilitate liver cancer progression (Yadav et al., 2020). This highlights the dual role of mechanotransduction in facilitating survival mechanisms, such as autophagy, while also enhancing aggressive phenotypes like increased invasiveness, contributing to cancer progression and metastasis.

These biomechanical interactions highlight the need for advanced computational modeling to decode complex mechanotransduction pathways and emerging technologies to precise quantification and prediction of mechanical alterations in tumors. Integrating these cutting-edge approaches with therapeutic strategies targeting mechanopathologies will offer new avenues for cancer treatment.

## 4 Computational modeling of mechanosensing

Mechanosensing, the capacity of cells and tissues to detect and respond to mechanical cues in their environment, is vital to processes such as development, wound healing, cancer progression, and tissue remodeling. As we mentioned previously, mechanical disturbances, including variations in stiffness and mechanical forces, influence cellular behavior, gene expression, and intercellular communication. While experimental advancements have significantly expanded our understanding of mechanosensing, the complexity of the underlying biomechanical processes demands computational methods to uncover the mechanisms driving cellular and tissue responses (Iskratsch et al., 2013; Pasapera et al., 2015). Computational modeling serves as a crucial tool for studying mechanosensing by combining experimental data with theoretical frameworks to simulate and predict cellular responses to mechanical stimuli. These mathematical models enable the quantification of mechanical perturbations’ effects on processes like mechanotransduction, cytoskeletal organization, and ECM interactions (Walcott and Sun, 2010; Schroer et al., 2014; Tao and Sun, 2015; Sun M. et al., 2016; Cheng et al., 2017; Jafarinia et al., 2024). Furthermore, they facilitate the investigation of conditions that are difficult to replicate experimentally, providing deeper insights into the principles of mechanobiology.

YAP and transcriptional coactivator with PDZ-binding motif (TAZ) known collectively as YAP/TAZ is accepted as a fundamental readout of cellular mechanotransduction. Specifically, YAP/TAZ mediates cellular responses to biomechanical stimuli like ECM stiffness, cell–cell contact, cytoskeletal stiffening, contractility and biochemical signals such as ECM composition and extracellular ligands (Dupont et al., 2011; Nardone et al., 2017; Koushki et al., 2023). Recently, YAP/TAZ has been shown to regulate cell proliferation, differentiation, apoptosis, migration, and inflammatory responses (Piccolo et al., 2013; Dupont, 2016; Kim H. B. et al., 2017; Choi et al., 2018). Sun M. et al. (2016) developed a mathematical model to investigate YAP/TAZ nuclear translocation in response to changes in ECM properties. The model extended existing frameworks of adhesion, RhoGTPase, and cytoskeleton dynamics to integrate cell adhesion, cytoskeleton behavior, and YAP/TAZ regulation. Incorporating F-actin, myosin, and LATS within an Ordinary Differential Equation model, it translated ECM ligand density and stiffness into a biochemical cascade initiated by FAK activation through a second-order Hill function - a suitable model for describing the high cooperativity of the adhesion molecules (Mitra et al., 2005; Provenzano et al., 2009). Scott et al. (2021) extended this model to investigate how cell shape and substrate dimensionality influence YAP/TAZ nuclear translocation. They have shown that YAP/TAZ activation and translocation relied on cytoskeletal dynamics and indirectly on its role in increasing active nuclear pores via Lamin A.

Cell microstructure influences differentiation, as demonstrated by Zeng et al.'s mathematical model of circular dorsal ruffles (CDRs)—actin-rich rings on growth factor-stimulated cells—showing that matrix rigidity impacts CDR size and lifespan through Rac-Rho antagonism (Zeng et al., 2011). The conversion of fibroblasts into myofibroblasts is significantly influenced by matrix rigidity. To investigate the mechanisms underlying the mechanical regulation of αSMA production, an ODE-based model was developed (Schroer et al., 2014). This model considered two primary inputs: growth factor signals, such as TGF-β and FGF, which activate downstream pathways like p38 and ERK, and matrix rigidity. Matrix rigidity was integrated into the model by incorporating the activity levels of intracellular kinases, including Src and FAK, which scale logarithmically with matrix stiffness. The model’s findings indicated that αSMA production is promoted by the activity of p38 and Src while being inhibited by ERK signalling, shedding light on the intricate balance of biochemical and mechanical inputs in regulating myofibroblast differentiation.

Computational modeling of mechanosensing is a valuable tool that deepens our understanding of biomechanical interactions, drives therapeutic advancements, and fosters innovation in both basic and applied biomedical research. However, there is a need to enhance computational approaches for mechanosensing and mechanotransduction, as current research lacks sufficient coverage in this area. Integrating these models with large-scale multiomics analyses, such as proteomics and transcriptomics, will offer a more comprehensive understanding of mechanotransduction dynamics and their impact on therapeutic efficacy.

## 5 Targeting mechanopathologies for cancer therapy

Standard-of-care therapies, even though they can effectively reduce tumor size or achieve temporary remission, they frequently fall short of providing a long-term cure, particularly in aggressive or advanced cancers (Torphy et al., 2018). Several strategies have been suggested to target mechanopathologies, focusing on restoring mechanical abnormalities in cellular and tissue level, to enhance cancer treatment (Martin et al., 2019a; Hyun and Kim, 2022; Liu et al., 2023). These treatments aim to normalize the abnormal mechanical properties of tumor cells and their surrounding mechanical environment. Mechanotherapies include targeting of i) ECM components or their production mechanisms, ii) cell contractions, iii) mechanosensors and iv) downstream mechanotransduction signalling pathways (Linke et al., 2024). Specifically, matrix proteins (e.g., LOX, CTGF, CD44) of TME can be targeted by inhibiting their production or remodelling or through enzymatic digestion (Saatci et al., 2020). CAFs population can be normalized by blocking sonic hedgehog (SHH) (Mpekris et al., 2017; Shroff et al., 2024), fibroblast activation protein (Xiao et al., 2023), TGFβ receptor (Panagi et al., 2020) or C-X-C motif chemokine receptor 4 (CXCR4) – C-X-C motif chemokine ligand 12(CXCL12) signalling (DiPersio et al., 2009; Chen et al., 2019). Cell contractility can be reduced via JAK-STAT, Rho-ROCK (Kim S. et al., 2021), or FAK-SRC inhibition (Haderk et al., 2024). For example, modulating Rho activity through targeting guanine nucleotide-exchange factors can affect Rho GTPases (Shang et al., 2012). Mechanosensors like caveolae, transient receptor potential cation channels (TRPs) (Nam et al., 2019), integrins and PIEZO 1/2 channels can be blocked, as well as their downstream pathways such as PI3K–AKT, β-catenin, JNK, YAP and p38-MAPK (Bergonzini et al., 2022; Monteiro et al., 2023). Specifically, PIEZO1/2 activation can be inhibited by non-specific blockers, such as spider toxin GsMtx4, ruthenium red, gadolinium ions and benzbromarone (Coste et al., 2010; Xiao, 2020; Bae et al., 2011; Liang et al., 2024). Normalizing mechanosensing with tropomyosin and myosin II, along with cytotoxic therapies or surgery to relieve solid stress, are also potential strategies (Kalli et al., 2022).

**FIGURE 2:**
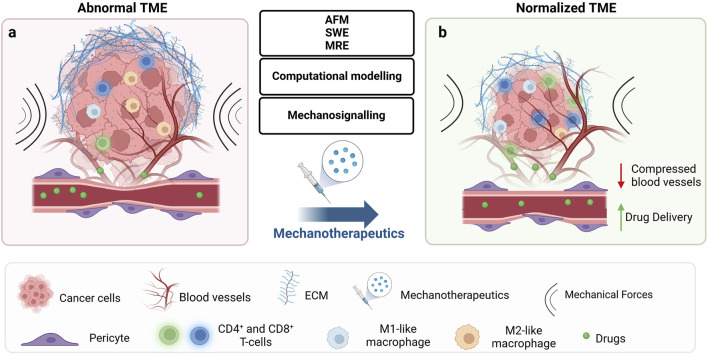
Mechanotherapeutics induce tumor microenvironment normalization and improve therapeutic outcomes. **(A)** The abnormal tumor microenvironment (TME) is characterized by high fibrosis and excessive extracellular matrix (ECM) deposition, including collagen and hyaluronan. This dense ECM leads to tumor stiffening and the buildup of mechanical forces within the tumor. These forces act at the cellular level to regulate signalling pathways implicated in key cellular responses, ultimately affecting treatment outcomes. At the tissue level, they compress blood vessels, reducing tumor blood flow and oxygenation, which results in hypoperfusion and hypoxia. The combination of low blood flow and dense ECM creates a physical barrier that not only impedes drug delivery and immune cell infiltration but also contributes to therapeutic resistance and tumor progression. **(B)** Emerging technologies, including methods for detecting the mechanical aspects of tumors at the tissue level (AFM, SWE, MRE), computational modelling, and identification of deregulated signalling mechanisms, could offer key insights into how biomechanically altered TME drive tumor progression and suggest potential therapeutic strategies to overcome them. These strategies include mechanotherapeutics, which typically involve existing medications used to influence the TME, or targeted therapies acting at the cellular level to modulate mechanosignaling. By normalizing the TME at the tissue level, pressure on blood vessels is relieved, improving drug delivery and oxygenation. Furthermore, improved perfusion induces immunostimulation by increasing levels of effector immune cells such as CD4^+^ and CD8^+^ T-cells and polarizing macrophages from an immunosuppressive M2-like phenotype to an immunosupportive M1-like phenotype. Created with BioRender.com.

Instead of blocking mechanotransduction pathways triggered by mechanical stress, addressing the root cause of solid stress can normalize the TME by directly reducing ECM components and reprogramming CAFs, thereby overcoming resistance to anticancer therapies (Nia et al., 2020; Jain et al., 2014; Mpekris et al., 2024a). Reducing solid stress can normalize blood vessels, improve perfusion and thus, enhance the delivery of chemotherapy, immunotherapy, and other treatments (Papageorgis et al., 2017; Polydorou et al., 2017; Panagi et al., 2020; Mpekris et al., 2017; Mpekris et al., 2021; Voutouri et al., 2021; Panagi et al., 2022; Mpekris et al., 2023a; Mpekris et al., 2024b; Panagi et al., 2024; Chauhan et al., 2013). Moreover, reducing solid stress levels can alleviate hypoxia and thus, stimulate immune responses, allowing immune cells to more effectively target and destroy cancer cells (Panagi et al., 2022; Mpekris et al., 2024b; Panagi et al., 2024; Mpekris et al., 2023b). Approved drugs (e.g., antihypertensive, anti-fibrotic, antihistamine) have been repurposed to achieve modulation of the mechanical TME, which we refer to as mechanotherapeutics (Mpekris et al., 2024a; Sheridan, 2019) (Figure 2). It might be argued that enhanced perfusion and increased delivery of oxygen and nutrients can support tumor progression. However, we have not seen this happening in several murine tumor types (Papageorgis et al., 2017; Mpekris et al., 2017; Voutouri et al., 2021; Panagi et al., 2024), and in addition mechanotherapeutics are not administered alone but in combination with anti-cancer to optimize therapeutic outcomes.

One example of such mechanotherapeutics is losartan, an anti-hypertensive and angiotensin receptor blocker, which was been successfully repurposed to improve therapeutic efficacy by decompressing blood vessels and enhancing perfusion in breast and pancreatic tumors in mice (Chauhan et al., 2013; Diop-Frimpong et al., 2011; Liu et al., 2012b). It was the first mechanotherapeutic to reach clinical trials, where its combination with FOLFIRINOX and radiation made 60% of previously unresectable pancreatic tumors resectable (Murphy et al., 2019). Losartan is now being tested with chemoradiation and the immune checkpoint inhibitor nivolumab for pancreatic cancer (NCT03563248). Its clinical success has established losartan as the “gold standard” for mechanotherapeutics. Another agent is ketotifen, an antihistamine drug that inhibits mast cell activation, suppresses CAF proliferation and ECM components in sarcomas (Mpekris et al., 2024b; Panagi et al., 2024; Charalambous et al., 2024). A phase II clinical trial is currently investigating its potential to enhance chemotherapy in sarcoma patients (EudraCT Number: 2022-002311-39). Additionally, previous studies successfully repurposed tranilast, an anti-fibrotic antihistamine approved in Japan and South Korea and pirfenidone, a globally approved anti-fibrotic drug for the treatment of idiopathic pulmonary fibrosis. These drugs have been shown to reduce solid and fluid stresses within tumors, improving perfusion and enhancing the effectiveness of chemotherapy, nanotherapy, and immunotherapy in both primary and metastatic sites (Papageorgis et al., 2017; Polydorou et al., 2017; Panagi et al., 2020; Mpekris et al., 2021; Panagi et al., 2022; Mpekris et al., 2023a). The underlying mechanism of the repurposed drugs involved suppression of TGF-β signalling and downregulation of ECM components like collagen and hyaluronan, which remodel the TME to improve drug delivery and immune response activation.

Other mechanotherapeutics under investigation include the antihyperglycemic agent metformin (Incio et al., 2015). Metformin suppressed the mTOR activity by activating LKB1 tumor suppressor and AMP-activated protein kinase (AMPK), inhibited protein synthesis, halted the cell cycle, induced apoptosis and autophagy through p53 and p21, lowered blood insulin levels, inhibited the unfolded protein response (UPR) and stimulated the immune system. It also targeted and eliminated cancer stem cells, prevented angiogenesis and decreased elevated lipid levels. Another drug that have been repurposed is the corticosteroid dexamethasone, which binds to the glucocorticoid receptor and regulate gene expression by inhibiting the production of inflammatory cytokines, suppressing activity of immune cells, promoting gluconeogenesis and inducing apoptosis (Martin et al., 2019b). Also, the endothelin receptor antagonist bosentan which inhibits the activity of endothelin-1 (Voutouri et al., 2021), a peptide that promotes tumor growth, angiogenesis and metastasis is being tested in a Phase I clinical trial for unresectable pancreatic cancer (Clinical-Trials.gov identifier: NCT04158635). Paricalcitol, targeting the vitamin D receptor on CAFs, is another agent of interest (Schwartz et al., 2008).

Combining drugs that target ECM components or mechanosignalling within tumors with existing cancer therapies is also a promising approach, as it addresses multiple aspects of cancer, enhancing therapeutic success and reducing the risk of drug resistance. Several clinical trials are exploring such combinations, including defactinib (FAK inhibitor) with pembrolizumab for pancreatic cancer (NCT03727880), cetuximab (collagen-targeting) with monalizumab in head and neck squamous cell carcinoma (NCT04590963), talabostat (fibroblast activation protein inhibitor) with pembrolizumab in advanced cancers (NCT04171219), and dasatinib (DDR2 inhibitor) with ipilimumab in gastrointestinal stromal tumors and sarcomas (NCT01643278) (He et al., 2021).

Combinational therapies can become more successful when combined with the knowledge offered from emerging technologies that provide critical insights into the mechanical aspects of cancer. The necessity to visualize and characterize the physical features of tumors on-the-spot, such as stiffness, adherence, and stiffness, can lead to the development of innovative tools for personalized therapy. The goal of utilizing these innovative technologies, is to bring increased awareness on the impact of the mechanical properties of tumors on drug response and cancer progression, thus optimizing therapeutic strategies.

## 6 Emerging technologies in tumor mechanopathological research

Emerging technologies are revolutionizing our ability to study the mechanical aspects of cancer, shedding light on how physical forces and structural abnormalities within tumors contribute to disease progression and therapy resistance. Specifically, cutting-edge technologies can drive mechanopathological research by enabling precise characterization and real-time visualization of mechanical properties, cellular interactions, and tissue architecture. By analyzing physical properties such as stiffness, adherence, and perfusion, researchers can gain critical insights into the behavior and characteristics of cancer cells and tumor tissues. These mechanical features allow investigators to determine the likelihood of cells being malignant, assess whether cancerous cells are invasive or capable of metastasizing, and evaluate the effects of therapeutic agents on tumor cells (Deng et al., 2018). Beyond these applications, such studies also help elucidate the broader biomechanical dynamics at play in diseased tissues.

Over the past decade, there has been a remarkable surge in interest in the mechanical analysis of biological specimens, particularly within the context of understanding and addressing various diseases. This growing focus underscores the importance of biomechanics as a complementary approach to traditional molecular and cellular biology in unraveling disease mechanisms and developing new treatment strategies (Najera et al., 2023). As a result, several approaches to examining tissue and cell mechanics have been refined and developed. Among these methods are (i) Atomic Force Microscopy (AFM) (Najera et al., 2023; Weber et al., 2022; Stylianou et al., 2022), (ii) Ultrasound Shear Wave Elastography (SWE) (Yoo et al., 2020; Park and Kang, 2021; Voutouri et al., 2023a; Togawa et al., 2024), (iii) Magnetic Resonance Elastography (MRE) (Yang and Qiu, 2021; Guo et al., 2023; Kim et al., 2024), (iv) microfluidics (Akgonullu et al., 2021; Mehta et al., 2022; Regmi et al., 2022) and (v) other emerging and less clinically translatable techniques that measure cellular and tissue mechanical, including tweezers, traction force microscopy, etc. Although these methods are by no means the only options.

### 6.1 Atomic force microscopy (AFM)

Atomic force microscopy (AFM) is widely used to analyze the mechanical properties of biological materials at high resolution. It has become a valuable tool in cancer research, linking mechanobiology with cancer initiation, progression, and treatment resistance (Plodinec et al., 2012; Stylianou et al., 2022; Tian et al., 2015; Stylianou et al., 2018b). While most AFM studies focus on cellular nanomechanics, recent efforts have extended to characterizing tumors and tumor-bearing tissues, offering new insights into cancer mechanopathology at the tissue level.

Several live-cell studies have explored the correlation between malignancy and cell deformability, measuring tissue stiffness (i.e., Young’s modulus) using AFM (Cross et al., 2011; Fuhrmann et al., 2011; Bastatas et al., 2012). AFM studies have been conducted on different cell lines, such as bladder (Lekka et al., 1999), breast (Li et al., 2008) and prostate (Faria et al., 2008) cancer cells, demonstrating that cancer cells are softer than normal cells. For instance, Lekka et al. (2012) demonstrated that cancer cells exhibit significantly greater deformability compared to healthy cells. Their study focused on three different prostate cancer cell lines and two breast cancer cell lines, revealing that these malignant cells have a markedly higher capacity for deformation (Lekka et al., 2012). Furthermore, numerous AFM studies focus on the nanomechanical characterization of cells to identify novel mechanical biomarkers (Najera et al., 2023).

AFM has been also employed to map stiffness profiles of normal, benign, and malignant human breast biopsy tissues. Normal and benign tissues showed unimodal profiles, while malignant tissues displayed bimodal profiles due to their heterogeneity. In malignant tissues, the lower elasticity peak corresponded to soft cancer cells, and the higher elasticity peak reflected the stiffer tumor stroma (Plodinec et al., 2012). In our previous studies, tumor stiffness has been measured from biopsies using AFM, which was able to identify unique Young’s modulus distribution defined as “nanomechanical fingerprints” for tumor and normal tissues and it was tested as a marker for tumor detection (Stylianou et al., 2018b; Stylianou et al., 2023). Our research supported the hypothesis that AFM-derived nanomechanical fingerprints are sensitive to TME modifications and could thus, be considered for predicting and monitoring TME normalization and efficacy of cancer therapy (Stylianou et al., 2022). Specifically, in murine tumor models, AFM was used to measure nanomechanical changes during tumor progression and treatment with the mechanotherapeutic tranilast and the chemotherapy doxorubicin. Nanomechanical data were correlated with *ex vivo* TME structural analysis, revealing that AFM can detect subtle changes in tumor nanomechanics during cancer progression and under different treatments. Tian et al. showed that human liver cancer cells have lower elasticity and ECM higher elasticity, distinguishing cancer from cirrhotic and normal tissues and aiding in predicting recurrence (Tian et al., 2015).

Generally, AFM has numerous advantages as it is a non-destructive method, applicable to live cells or tissues, which provides quantitative, nanoscale data of stiffness and viscoelasticity but it requires a biopsy to be obtained for analysis, it might not account for the spatial heterogeneity of tumors and AFM imaging is not available in oncology centers.

### 6.2 Ultrasound shear wave elastography (SWE)

Ultrasound Shear Wave Elastography (SWE) is an advanced, non-invasive imaging technique used to assess the mechanical properties of tissues by acquiring elasticity maps and measuring their stiffness (Panagi et al., 2022; Mpekris et al., 2023a; Voutouri et al., 2023a; Cosgrove et al., 2012; Lee et al., 2015; Singh et al., 2021). It has gained prominence in medical diagnostics, particularly for its ability to differentiate between healthy and pathological tissues in real time, such as in liver fibrosis. By utilizing acoustic waves, SWE provides a quantitative evaluation of tissue elasticity, offering valuable insights for the diagnosis and management of various diseases.

SWE-derived stiffness measures by accounting for average values of the elastic modulus over the entire tumor region or by applying machine learning methods to identify complex patterns and subvisual features have been used not only for cancer detection (Zhang et al., 2016) but also for the prediction of tumor response to therapy (Voutouri et al., 2023a; Voutouri et al., 2023b; Englezos et al., 2024). It has been demonstrated that SWE-measured tumor stiffness correlated with prognostic indicators like immunohistochemical profiles, molecular subtypes, and lymphovascular invasion in breast cancer (Lee et al., 2015; Youk et al., 2013; Lee S. H. et al., 2014; Au et al., 2015; Youk et al., 2017). Yoo et al. conducted a detailed investigation into the relationships between quantitative stiffness parameters measured with SWE and key tumor characteristics, including hypoxia and histologic prognostic biomarkers, in invasive breast cancer tissues from female patients (Yoo et al., 2020). Their findings revealed that tumor stiffness was significantly correlated with levels of tumor hypoxia as well as several histologic biomarkers that are critical for understanding tumor behavior and progression. Importantly, their analysis highlighted that average tissue elasticity held independent prognostic value for assessing tumor hypoxia in multivariable analysis. In a recent clinical study, Togawa et al. evaluated the role SWE in axillary staging for patients undergoing initial breast cancer diagnostics (Togawa et al., 2024). They have shown that lymph node metastases assessed with SWE showed significantly higher elasticity values compared to benign lymph nodes.

These findings suggest that SWE can serve as a powerful noninvasive imaging modality capable of predicting tumor prognosis and assisting pretreatment risk stratification for cancer patients.

### 6.3 Magnetic resonance elastography (MRE)

Magnetic Resonance Elastography (MRE) is an advanced and versatile imaging technique that allows for the detailed mapping of the mechanical properties of soft biological tissues, particularly their stiffness or viscoelasticity, by measuring how tissues deform in response to mechanical waves (Muthupillai et al., 1995). Originally developed for detecting liver fibrosis, MRE has gained widespread use in medical practice and is increasingly being applied in the diagnosis and monitoring of a broad range of diseases, including chronic kidney diseases and various forms of cancer (Svensson et al., 2023; Fels-Palesandro et al., 2024). In a clinical study, Venkatesh et al. used MRE to evaluate 29 patients with 44 liver tumors, finding that malignant liver tumors had higher stiffness than benign tumors, liver fibrosis and normal liver parenchyma with 5 kPa identified as the critical threshold distinguishing malignant from benign or normal tissue (Venkatesh et al., 2008). MRE also employed for tumor detection in patients with prostate cancer and it was found that tumor stiffness was significantly higher from normal parenchyma and thus, could be used for differentiated prostate cancer from benign prostate hyperplasia (Kim et al., 2024). MRE enhanced the diagnostic accuracy of MRI for breast cancer by addressing the overlap in elasticity between benign and malignant tumors, as MRI alone cannot reliably differentiate them; combining MRI with viscoelastic parameters (elasticity and viscosity) enabled a more comprehensive evaluation (Sinkus et al., 2007; Siegmann et al., 2010; Balleyguier et al., 2018). In brain tumors, the difficulty of tumor resection is significantly influenced by tumor consistency, with studies showing that MRE-measured stiffness of meningiomas and pituitary adenomas closely correlates with surgeons’ subjective assessments of tumor consistency during surgery (Murphy et al., 2013).

Recently, MRE was performed to monitor response to immunotherapy in orthotopic syngeneic experimental glioma (Streibel et al., 2024). Specifically, MRE has demonstrated potential as a promising non-invasive imaging technique for monitoring immunotherapy by measuring changes in tumor mechanics associated with treatment response. MRE has also been used in the diagnosis of colorectal cancer, and thyroid tumors (Juge et al., 2012; Gregory et al., 2018). In conclusion, research indicates that MRE effectively differentiates between benign and malignant tumors, enhancing the specificity and sensitivity of tumor diagnosis.

### 6.4 Microfluidics

Microfluidics, a cutting-edge technology that manipulates fluids in microscale channels, has emerged as a transformative tool in studying tumor mechanopathology. This approach allows researchers to recreate and analyze the complex mechanical and biochemical conditions of the TME in highly controlled settings, providing valuable insights into cell migration, invasion and treatment resistance (Mehta et al., 2022; Hou et al., 2009; Lee H. et al., 2014; Nagaraju et al., 2018; Yang et al., 2022). Furthermore, enables researchers to study the impact of mechanical forces, such as interstitial fluid pressure on drug delivery and model tumor-vascular interactions by developing organ-on-a-chip models (Zervantonakis et al., 2012; Wang and Wang, 2014; Skardal et al., 2016; Nashimoto et al., 2020; Wang et al., 2020).

Hou et al. studied the deformability of benign (MCF-10A) and nonmetastatic (MCF-7) breast tumor cells, measuring parameters like cell velocity, transit time, and deformation (Hou et al., 2009). While both cell types showed similar transit velocities, suggesting equivalent friction against microfluidic walls, MCF-10A cells exhibited longer entry times than MCF-7 cells indicating that MCF-10A cells are stiffer and less deformable than MCF-7 cells. To study the metastatic cascade of invasion, intravasation and extravasation of metastatic and nonmetastatic cell lines and test inhibitors to block cancer cell invasion, a microfluidic chip with two compartments: one for 3D cancer cell culture in Matrigel (intravasation) and another for detecting metastasized cells via epithelial adhesion molecules (extravasation) was employed (Shin et al., 2011). Zervantonakis et al. (2012) developed a 3D microfluidic platform to model the tumor-vascular interface, linking cytokine-induced endothelial activation, macrophage signaling, and increased endothelial permeability with enhanced intravasation, which could be reduced by blocking TNF-α to restore barrier integrity. Song and Munn (2011) using a microfluidic tissue model of angiogenic sprouting, they found that fluid shear stress, suppressed endothelial cell sprouting through a nitric oxide-dependent mechanism, while interstitial flow from extravasating plasma directed endothelial organization and sprout formation. Additionally, they recapitulated the dynamics of vascular anastomosis and they demonstrated that convective flow through the 3-D ECM enhanced the rate of VEGF-induced anastomosis compared to static conditions (Song et al., 2012). Several studies have demonstrated that tumor spheroids (employed instead of single cells), responded to drugs like doxorubicin (Yu et al., 2010) and vincristine (Wang and Wang, 2014) in a dose-dependent manner.

### 6.5 Emerging and less clinically translatable techniques for measuring cellular and tissue mechanics

Apart from AFM, which allows precise quantification of cell stiffness, and the forces exerted on substrates, micropipette aspiration (Mitchison and Swann, 1954) and optical- (Ashkin et al., 1986), magnetic- (Bausch et al., 1999) and acoustic (Wu, 1991)- tweezers, enable force measurement by aspirating cells or manipulating ECM components. Microplate actuators (Foty et al., 1994) also allows for understanding of cell and ECM interactions, while techniques such as Brillouin microscopy (Brillouin, 1922) and tissue dissection and relaxation (Moore and Burt, 1939) measure elastic properties, i.e., viscoelasticity. In addition to these technologies, other techniques have been developed to measure tissue stress *in vitro* and *ex vivo*, beginning with those designed for 2D cell cultures. These methods are commonly employed to study cell-ECM interactions and the mechanical forces exerted by cells on their environment in flat, 2D settings. Key techniques include 2D Traction Force Microscopy (TFM) (Dembo and Wang, 1999; Schwarz and Soine, 2015; Style et al., 2014), which measures the forces exerted by cells on a flexible substrate; micropillar arrays, where cells deform tiny pillars to quantify forces; and monolayer stress microscopy (MSM) (Tambe et al., 2013), which visualizes local stress distributions in cell monolayers. Additionally, tensile tests on cultured tissues are introduced, where tissue samples are mechanically stretched to measure their stress-strain properties (Harris et al., 2012; Merzouki et al., 2016). Techniques like 3D TFM (Legant et al., 2010; Steinwachs et al., 2016), extend the 2D TFM approach into three-dimensional matrices, providing insights into how cells generate and respond to forces in a 3D context. The microbulge test (Latorre et al., 2018), another method for assessing mechanical properties in 3D cultures, involves observing the deformation of a substrate when subjected to cellular forces. These techniques allow for a deeper understanding of how cells behave within more physiologically relevant environments, such as tissue constructs, tumors, or organoids.

Although measuring stresses *in vivo* remains a challenge, techniques available include servo-null methods (Navis and Bagnat, 2015; Wiederhielm et al., 1964), that measure stress by detecting displacements in a tissue under mechanical loading, while inclusions (Girardo et al., 2018) (small, stiff objects embedded within tissues) can serve as markers to monitor mechanical forces by tracking their displacement. Förster resonance energy transfer (FRET) tension sensors serve as molecular tools that provide real-time measurement of forces at the cellular level by detecting changes in fluorescence intensity in response to mechanical tension (Gayrard and Borghi, 2016; Yasunaga et al., 2019). Additionally, laser ablation involves the use of a focused laser to cut tissue and observe the subsequent tissue movement to infer mechanical stresses (Hutson et al., 2009; Ma et al., 2009).

In summary, imaging modalities provide valuable mechanopathological data that enhance our understanding of tumor behavior, improve diagnostics and treatment planning, and contribute to better patient outcomes.

## 7 Conclusion

As most solid tumors exhibit abnormal biomechanical properties, understanding how these mechanical changes drive tumor progression and influence therapeutic responses is crucial. Mechanical alterations within the TME not only impair the delivery of therapeutic agents and immune cells but also activate mechanotransduction pathways that regulate key cellular functions, ultimately promoting tumor growth, metastasis, and therapy resistance.

Emerging technologies, such as advanced methods for detecting biomechanical abnormalities at the tissue level, large-scale multiomics for characterizing deregulated molecular mechanisms in tumor cells, and computational modeling, offer powerful tools for exploring the complex interactions between mechanical forces and tumor responses. Integrating these approaches can facilitate the development of combined therapies aimed at restoring the TME and directly modulating mechanotransduction pathways in tumor cells (Figure 2). By employing these methodologies, we can refine current treatment strategies to incorporate tumor mechanobiology, ultimately leading to more effective, personalized therapies that address tumor complexity and improve patient outcomes.
